# The Human Will

**Published:** 1890-06

**Authors:** 


### THE HUMAN WILL.

There has appeared in London a certain mysterious French Count whose mission is to illustrate mechanically the power of the human will. His apparatus is thus described: "Upon a reel is wound a length of silver wire, measuring 75,000 metres. Two magnetic needles crossing each other in a contrary direction are fixed upon the reel, and suspended by a slender thread. The apparatus works under glass, like a watch, so that no tampering with the mechanism is possible. It is mounted, moreover, on a high stand. The Count takes hold of the two conductors, to which are attached the two ends of the silver thread rolled upon the reel, and bids you order the machine to move to right or left, according to your will. Under this power alone, hitherto misunderstood or underrated—this, the mightiest power in the universe, according to Count P., the power of the Human Will—the machine will act without the contact of touch. To right or to left will the reel revolve, according to the fancy of the visitor. Without speech, without touch, by the mere mental influence alone, will the machine move in obedience to the unexpressed command. But not in all cases does the machine answer unreservedly. It is to the powerful will alone—the concentrated and fixed determination that it can be made to reply." It is stated that the Count has been converted from materialism "to the highest degree of religious faith, to conviction of the lofty destinies of man, and his connection with Divinity," and all this, apparently, because he reproduces experiments very familiar to Spiritualists.—*Light, London.*

